# Bluetongue in China: Current Status of Viruses, Vectors, Detection Methods, and Vaccines

**DOI:** 10.1155/tbed/5538034

**Published:** 2026-02-19

**Authors:** Jige Xin, Xincheng Ji, Zhigang Song, Weidong Zuo, Shanglian Yin, Yong Peng, Miao Ren, Jun Ai, Diangang Han

**Affiliations:** ^1^ Yunnan Key Laboratory of Veterinary Etiological Biology, College of Veterinary Medicine, Yunnan Agricultural University, Kunming, 650201, China, ynau.edu.cn; ^2^ Research Center for International Inspection and Quarantine Standard and Technical Regulation, General Administration of Customs, Beijing, 100013, China; ^3^ Animal Quarantine Laboratory, Technology Center of Mengla Customs, Mengla, 666300, China; ^4^ Animal Quarantine Laboratory, Technology Center of Kunming Customs, Kunming, 650200, China; ^5^ Animal Quarantine Laboratory, Technology Center of Ruili Customs, Ruili, 678600, China

**Keywords:** bluetongue, bluetongue virus, *Culicoides*, diagnostics, ruminants, vaccines

## Abstract

Bluetongue (BT) is a vector‐borne viral disease caused by the bluetongue virus (BTV), which can affect a variety of wild and domestic ruminants. Due to its significant impact on ruminant health and national economies, BT is classified as a notifiable multispecies disease by the World Organization for Animal Health (WOAH). In China, BT is listed as a Class II multispecies animal disease. This article provides a comprehensive review of the distribution of BTV and its primary insect vector, *Culicoides*, in China. Since BTV was first reported in China in 1979, BTV antibody‐positive samples have been detected in most parts of the country, with a total of 17 serotypes of BTV isolated (BTV‐1, 2, 3, 4, 5, 7, 9, 11, 12, 14, 15, 16, 17, 20, 21, 24, and 29). *Culicoides* are widely distributed across China. Currently, studies have been conducted on climatic factors influencing their distribution and blood‐sucking habits. To improve the efficiency of BTV detection in China, various detection methods have been explored, including polymerase chain reaction (PCR), loop‐mediated isothermal amplification (LAMP), bio‐bar code assay (BCA) for viral detection, as well as enzyme‐linked immunosorbent assay (ELISA), agar gel immunodiffusion (AGID) and colloidal gold immunochromatography test strips for antibody detection. Additionally, inactivated vaccines, attenuated vaccines, and recombinant vaccines were also investigated. This review summarizes the current knowledge on BTV vectors, viruses, and surveillance, as well as the development of BT vaccines in China. In light of the current situation of BT in China, it proposes comprehensive prevention and control recommendations, including enhancing awareness of the hazards of BT, implementing an integrated prevention and control technology system, and strengthening research related to BT prevention and control.

## 1. Introduction

### 1.1. Overview of Bluetongue (BT) Disease

BT is an infectious, non‐contagious viral disease transmitted by vectors, which is caused by the BT virus (BTV). It can infect a variety of wild and domestic ruminants, including sheep, goats, cattle, and buffaloes, as well as deer, pronghorn antelope, bighorn sheep, elephants, and camelids [1, 2]. BTV is naturally transmitted to susceptible hosts mainly through the bites of insects belonging to the genus *Culicoides* [3]. The insect vector serves a critical function in the transmission of BTV between animals, as these vectors acquire the virus through ingesting blood from infected hosts. Additional documented transmission pathways include venereal spread via semen, contact and oral transmission, transplacental transmission arising from uterine infections, and mechanical vector‐mediated transmission [4–6]. Depending on the serotype of the virus, the species of the infected animal, and whether the disease is the first epidemic or not, the clinical symptoms of the animals infected with BTV can vary from asymptomatic, subclinical symptoms, or even death. The common clinical symptoms of the disease include cyanosis of the tongue, fever, excessive salivation, lameness with coronitis, respiratory distress and sometimes lead to abortion and stillbirth of pregnant females [7, 8]. Sheep are the most susceptible to the disease and exhibit the highest morbidity and mortality rates following infection. In highly susceptible sheep populations, morbidity can reach up to 100%.

Given the potential for BTV infection in animals to be complicated by secondary bacterial or viral infections, certain susceptible breeds of sheep have even exhibited a mortality of 100% [9]. Cattle are usually asymptomatic carriers after being infected with BTV, but can be a potential virus reservoir and source of infection on the farm [10]. However, outbreaks of BTV‐8 in Europe during 2006 caused clinical disease in cattle [11]. BTV infection causes serious direct and indirect economic losses, including decreased growth and reproductive performance, as well as deaths and losses caused by early culling. Indirect losses include losses due to trade restrictions, expenditures on vaccination, diagnosis, vector control, and treatment [12, 13]. The 1996 BTV outbreak incurred an estimated economic loss of $3 billion worldwide [9]. Between 2006 and 2008, BTV‐8 infected over 24,000 animals in Germany [14], resulting in national‐level damages ranging from 157 to 203 million euros [12]. In 2014, the estimated cost of BTV in the US beef industry amounted to $95 billion [7]. Due to its significant impact on ruminant health and economy, BT is classified as a notifiable multispecies disease by the World Organization for Animal Health (WOAH) [15]. In China, BT is listed as a multispecies Class II animal disease (http://www.xmsyj.moa.gov.cn/gzdt/202206/t20220629_6403635.htm accessed on 15 October 2023).

### 1.2. Structure and Classification of BTV

BTV is a non‐enveloped virus belonging to the *Orbivirus genus* within the Reoviridae family. Its genome consists of 10 double‐stranded RNA (dsRNA) segments, which encode the following proteins: Seg‐1/VP1, Seg‐2/VP2, Seg‐3/VP3, Seg‐4/VP4, Seg‐5/NS1, Seg‐6/VP5, Seg‐7/VP7, Seg‐8/NS2, Seg‐9/VP6, NS4, Seg‐10/NS3, and NS3a [16]. As the least conserved viral protein, its sequence varies significantly among serotypes—for example, sequence identity ranges from 22.7% (between BTV‐4 and BTV‐20) to 72.9% (between BTV‐6 and BTV‐22) [17]. The variability of VP5 is second only to VP2, which can affect the production of BTV serotype‐specific antibodies by affecting the conformation of VP2 [18]. VP7 determines the group specificity of BTV, with amino acid sequence similarities of up to 94% among VP7 proteins of different BTV serotypes [19]. The phylogenetic tree constructed by each gene segment of BTV showed that the isolated BTV strains in different countries/regions were divided into two regional types: Eastern topotype and Western topotype [20]. According to WOAH Terrestrial Manual 2021, there are 27 recognized BTV serotypes including BTV‐25, which was detected in Switzerland in 2007 [21]; BTV‐26, isolated in Kuwait in 2010 [22]; BTV‐27, isolated in France in 2014 [23]. In recent years, several putative novel atypical BTV serotypes have been reported [24–33]. The serum neutralization methods were insufficient for accurately typing all these novel atypical strains, thus making it impossible to assign them to a specific serotype. Ries et al. [27, 28] categorized the “putative novel atypical serotypes” based on their segment‐2 sequence identities and the time point of sampling and identified a total of 9 putative BTV serotypes. Among them, BTV‐28/1537/14 and SPvvvv/03 were classified as BTV‐28, SPvvvv/02 as BTV‐29, BTV‐X/XJ1407 and BTV‐MNG2/2016 as BTV‐30, V196/XJ/2014 as BTV‐31, BTV‐X ITL2015 and BTV‐X ITL2015 strain 33 531 as BTV‐32, BTV‐MNG3/2016 as BTV‐33, BTV‐Y/TUN2017 as BTV‐34, BTV‐MNG1/2018 as BTV‐35, and BTV‐36‐CH0219 as BTV‐36. Isolation details of some known serotypes of BTV are provided in Table 1.

**Table 1:** Isolation details of the known serotypes of BTV [25–28, 34].

Serotypes	Year	Origin	Species isolated from
BTV‐4	1900	Cape Province—South Africa	Sheep
BTV‐8	1937	Onderstepoort—South Africa	Sheep
BTV‐12	1941	Beaufort‐West—South Africa	Cattle
BTV‐9	1942	Pretoria—South Africa	Sheep
BTV‐3	1944	Cyprus	Sheep
BTV‐11	1944	Beaufort‐West—South Africa	Sheep
BTV‐5	1953	Machadodorp—South Africa	Sheep
BTV‐7	1955	Utrecht—South Africa	Sheep
BTV‐10	1956	Portugal	Sheep
BTV‐1	1958	Biggarsberg—South Africa	Sheep
BTV‐2	1958	Vryheid—South Africa	Sheep
BTV‐6	1958	Vryheid—South Africa	Sheep
BTV‐13	1959	Transvaal/Natal—South Africa	Unknown
BTV‐14	1959	Transvaal/Natal—South Africa	Unknown
BTV‐16	1959	West Pakistan	Sheep
BTV‐15	1960	Ermelo district—South Africa	Cattle
BTV‐17	1962	United States	Unknown
BTV‐20	1975	Australia	*Culicoides*
BTV‐18	1976	Republic of South Africa (RSA)	Sheep
BTV‐19	1976	RSA	Sheep
BTV‐21	1979	Australia	Cattle
BTV‐23	1982	Australia	Cattle
BTV‐22	1992	RSA	*Culicoides*
BTV‐24	1992	RSA	Sheep
BTV‐25	2007	Toggenburg—Switzerland	Goats
BTV‐26	2010	Kuwait	Sheep
BTV‐27	2014	Corsica, France	Goats
BTV‐28 (BTV‐28/1537/14)	2014	Israel	Sheep‐pox vaccine
BTV‐29 (SPvvvv/02)	2014	Israel	Sheep‐pox vaccine
BTV‐30 (BTV‐X/XJ1407)	2014	Xinjiang, China	Goats
BTV‐31 (V196/XJ/2014)	2014	Xinjiang, China	Goats
BTV‐32 (BTV‐X‐ITL2015)	2015	Italy	Goats
BTV‐33 (BTV‐MNG3/2016)	2016	Mongolia	Sheep and goats
BTV‐34 (BTV‐Y/TUN2017)	2017	Tunisia	Sheep
BTV‐35 (BTV‐MNG1/2018)	2018	Mongolia	Goats
BTV‐36 (BTV‐36‐CH0219)	2019	Mongolia	Goats

*Note*: In the study conducted by Ries et al. [28], BTV‐29 corresponds to SPvvvv/02, while BTV‐31 corresponds to V196/XJ/2014. However, in the study by Yang et al. [33], V196/XJ/2014 is referred to as BTV‐29. To avoid any confusion, it should be clarified that in the following context, BTV‐29 specifically refers to V196/XJ/2014.

### 1.3. Global Epidemiology of BT

**Figure 1:**
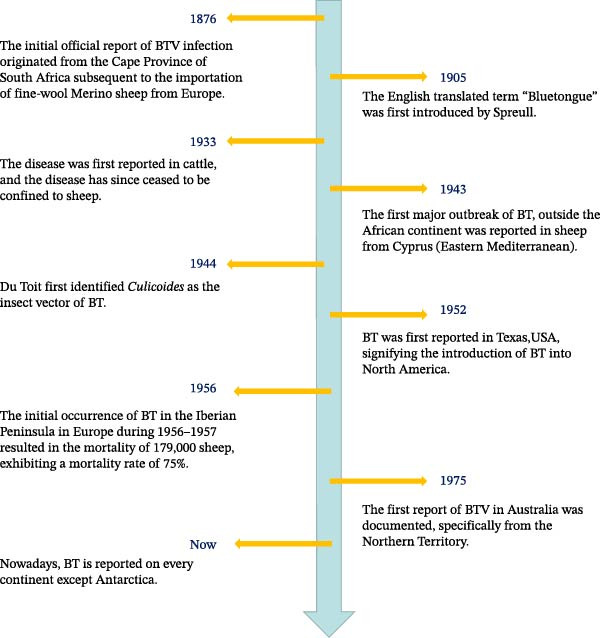
Timeline‐chronological identification of bluetongue (BT) in the world. Data from Maclachlan [36], Spreull [37], Vellema [38], Rivera et al. [39], and White et al. [40].

BT was first documented in South Africa in 1876, and BTV was first detected among merino wool sheep in South Africa in 1905. However, the extensive geographical expansion of vector habitats and the movement of animals through trade led to a distribution of BTV worldwide. Currently, the disease has been reported on all continents except Antarctica [7, 35] (Figure 1).

Currently, BTV is primarily distributed in tropical, subtropical, and temperate regions, spanning latitudes between 40°N and 35°S [38]. However, the distribution range of BT extends further north in some countries: in the United States and China, BT has been documented as far as ~50°N [41]. BTV‐3 was detected from sheep in Germany by real‐time polymerase chain reaction (PCR) in October 2023, which is also the most recent case reported by WOAH (https://wahis.woah.org/#/event-management accessed on 30 September 2023). In recent years, the global increase in temperature, accompanied by an increase in the movement of animals and goods, the emergence of new vector insects, and the gradual expansion of suitable geographical ranges for their survival have collectively contributed to the widespread prevalence of BTV. Additionally, the emergence of reassortant viruses through gene segment reassortment and the appearance of new serotypes or more pathogenic strains in certain regions have significantly heightened the threat posed by BT to global cattle and sheep farming.

## 2. Serological Surveillance of BTV in China

China boasts an extensive scale of cattle and sheep breeding, with 102.16 million heads of cattle and 326.27 million sheep recorded in 2022 (http://zdscxx.moa.gov.cn:8080/nyb/pc/search.jsp accessed on October 14, 2023). In addition, China’s cross‐border trade of cattle and sheep is substantial, indicating a consistent net import trend. According to data released by the General Administration of Customs, the value of China’s cattle imports in 2022 amounted to 804.3 million USD, while sheep imports were valued at 8.4 million USD. (http://stats.customs.gov.cn/ accessed on October 14th, 2023). China features an extensive territory, complex natural environment, and a wide distribution of diverse arbovirus transmission vectors. The emergence of novel BTV serotypes will potentially impact the sound development of China’s cattle and sheep industry, as well as international trade of livestock products. Notably, the BTV can infect a variety of wild and domestic ruminants, including sheep, goats, cattle, buffaloes, deer, and camelids, all of which are present in China. Therefore, conducting a systematic serological investigation on BTV is of utmost importance.

**Figure 2:**
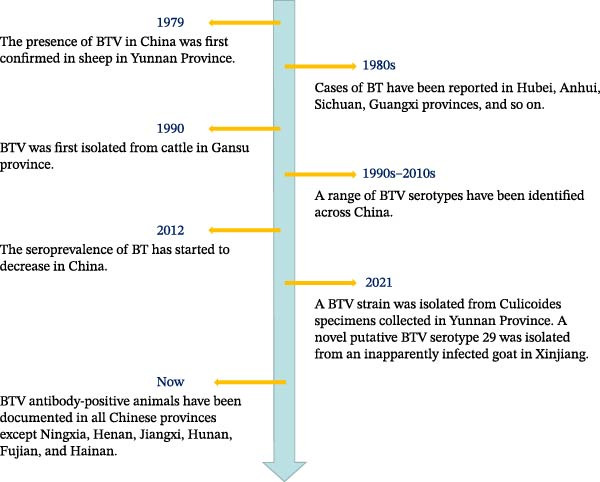
Timeline‐chronological development of BT in China.

The existence of BTV in China was first demonstrated by Zhang et al. [42] in 1979, specifically in Shizong County, Yunnan Province. Key milestones in the progression of BT are presented in Figure 2. The presence of BTV antibody‐positive samples has been detected in most regions across China, indicating a wide distribution of BTV throughout the country. This includes areas ranging from the westernmost Xinjiang Uygur Autonomous Region to the majority of eastern coastal provinces, as well as extending from the northernmost Heilongjiang Province to southern regions such as Yunnan Province, Guangxi Province, and Guangdong Province. China comprises 34 provincial‐level administrative regions, which include 23 provinces, 5 autonomous regions, 4 municipalities directly under the Central Government, and 2 special administrative regions. To date, BTV antibody‐positive animals have been identified in 18 of the 23 provinces, 4 of the 5 autonomous regions, and 3 of the 4 municipalities [43, 44]. Currently, there have been no reported cases of BTV antibody‐positive samples in Ningxia Hui Autonomous Region, Henan Province, Hunan Province, Jiangxi Province, and Fujian Province. However, it is important to note that these regions are surrounded by provinces where BTV antibody‐positive animals have been detected. Moreover, these regions possess the necessary climatic conditions for BT prevalence. Therefore, conducting serological testing on ruminants in these areas is imperative to ascertain the prevalence of BT.

Numerous reports have been published on the surveillance of BTV antibodies across different regions and time periods in China. To understand the prevalence and distribution of BT in the country, Zhu et al. [43] investigated and analyzed a total of 19,237 serum samples from 62 regions in 12 provinces, which were selected as representative based on climate and altitude factors, spanning from 2015 to 2017. The findings revealed that BTV antibody‐positive samples were widespread across all surveyed regions, exhibiting an average positive rate ranging from 4.33% to 38.14%. Guangxi, Chongqing, and Yunnan emerged as the top three regions with the highest BTV antibody‐positive rates. Conversely, Liaoning, Jilin, and Hebei exhibited the lowest positive rates. The seropositive rate tended to be higher in low latitude areas compared to high latitudes (ranging from 4.33% to 38.14%), while areas abundant in vegetation displayed higher seropositive rates than other areas within the same latitude range. By quarter, the third quarter (July–September) demonstrated the highest positive rate ranging between 23.3% and 46.7%, whereas the first quarter (January–March) exhibited the lowest positive rate ranging from 0% to 13.3%. These results indicate a close correlation between BTV prevalence and vector activity.

Yunnan Province is situated on the southwestern border of China, where the first discovery of BTV occurred, and it also harbors the highest diversity of BTV serotypes among all provinces. The region shares borders with Myanmar, Laos, and Vietnam. The prevailing hot and humid climate, coupled with extensive animal trade, creates favorable conditions for the transmission of BTV. Xie et al. [45] conducted a study in 2022 where they collected 1820 serum samples from nine border counties of Yunnan Province. The findings revealed a widespread prevalence of BTV in the border regions of Yunnan Province, with an escalating trend in infection. The increasing risk of pathogen introduction into China through foreign cattle necessitates more serious measures for prevention and control. Additionally, with climate warming, there is a gradual expansion in the geographical range of vector insects such as *Culicoides*. Furthermore, frequent animal movement and goods exchange activities have facilitated the progressive spread of BT to higher latitude areas.

Heilongjiang Province is situated in the northernmost region of China and is characterized by a frigid climate. Li et al. [46] detected 997 serum samples from cattle and sheep in various seasons across different regions of Heilongjiang Province between 2020 and 2021. The study revealed that the prevalence of BTV antibodies in bovine and sheep serum was found to be 13.70% and 12.65%, respectively, within this area. Additionally, Li et al. [46] research led to the identification of a BTV epidemic strain classified preliminarily as BTV‐1 type, indicating the presence of BTV even in China’s northernmost province.

## 3. Isolation of BTV in China

**Figure 3:**
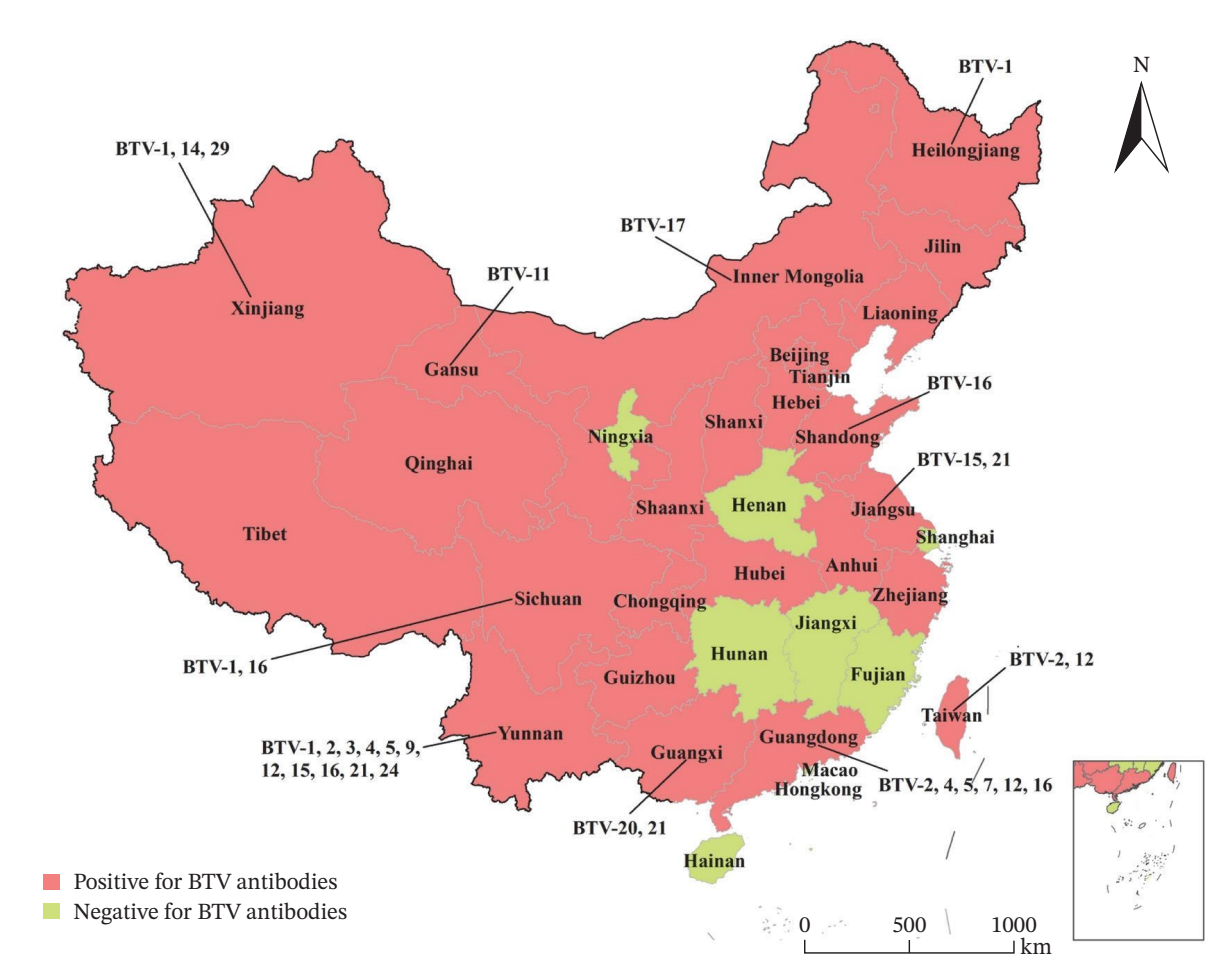
Review of BTV antibody surveillance and serotype isolation in China. Data from Li et al. [44, 46], Wang et al. [47] and Qi et al. [48]. Note: The red color indicates the detection of BTV antibody positive samples in the province/region, while the green color signifies no detection of BTV antibody positive samples. “BTV‐” indicates isolation of the corresponding serotype of BTV in the province/region. The results depicted in the figure represent a retrospective summary of diverse surveillance outcomes over an extended period and do not reflect the current bluetongue situation in each province/region.

By isolating BTV strains of different serotypes and subsequently sequencing their complete genomes, we can not only identify the prevalent local serotypes of BTV and enhance our understanding of globally diverse BTV strains but also facilitate the tracing of different serotype BTV strain origins and deepen our comprehension of BTV gene reassortment and diversity. Since the first strain of BTV was isolated in 1979, China has subsequently identified 17 serotypes of BTV from 11 provinces (autonomous regions and municipalities directly under the Central Government) [44, 46–48] (Figure 3).

Among them, BTV‐16 and BTV‐1 are the predominant serotypes circulating in China. The majority of serotypes were isolated from Yunnan Province, encompassing BTV‐1, ‐2, ‐3, ‐4, ‐5, ‐9, ‐12, ‐15, ‐16, ‐21, and ‐24. The reporting of only BTV antibody‐positive samples without BTV isolation and identification is still prevalent in numerous provincial administrative regions across China. Consequently, despite the isolation of 17 serotypes of BTV within China, the potential existence of additional serotypes cannot be disregarded. Yunnan Academy of Animal Husbandry and Veterinary Sciences not only reported the first occurrence of BT in China but also conducted extensive subsequent research, including BTV isolation, gene sequencing, and genetic characteristics analysis. From 1996 to 2017, they isolated a total of 12 serotypes (BTV‐1, ‐2, ‐3, ‐4, ‐5, ‐7, ‐9, ‐12, ‐15, ‐16, ‐21, and ‐24) of BTV strains from various regions in China [49]. BTV was predominantly detected in blood or tissue samples; however, Wang et al. [50] were able to isolate a BTV‐1 strain from *Culicoides* collected in Yunnan Province by detecting nucleic acid fragments and confirming through VP7 gene sequencing and indirect immunofluorescence testing. This discovery marks the first isolation of BTV from *Culicoides* in China, highlighting the need for strengthened vector surveillance measures in BT prevention and control.

**Figure 4:**
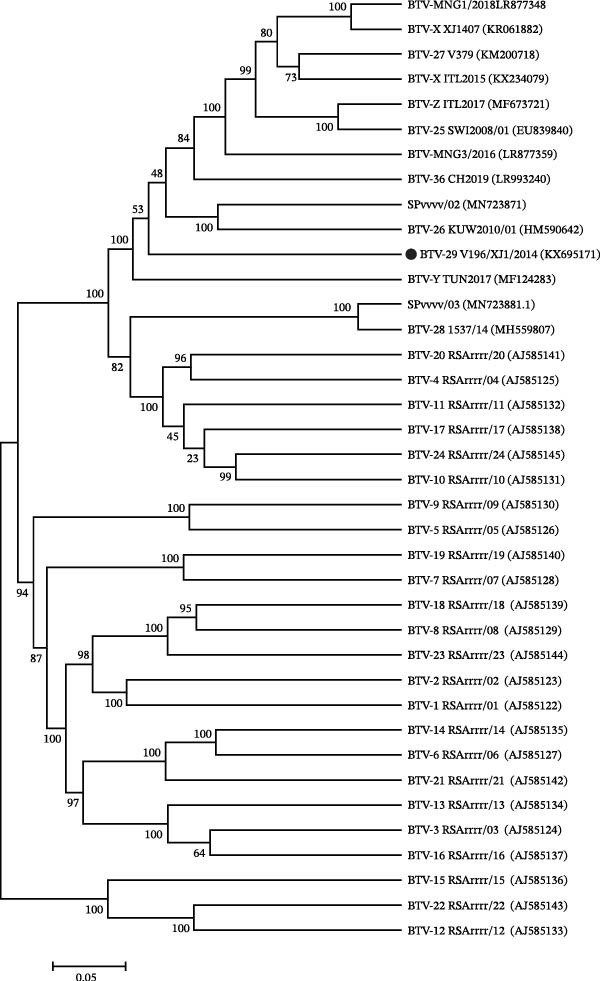
Phylogenetic relationships based on Seg‐2 of V196/XJ/2014 with reference strains of recognized bluetongue virus serotypes.

The annual import and export trade of cattle and sheep in China is substantial, with BTV isolation also in the entry‐exit animal quarantine process. Zhang et al. [51] successfully isolated BTV from antelopes imported from the United States in 1988 and sheep imported from the Soviet Union in 1989, marking the first instance of BTV isolation from imported animals in China. However, the serotype of the isolated BTV was not determined at that time. Yang et al. [33] conducted a comprehensive analysis including BTV pan‐qRT‐PCR, AC‐ELISA testing, viral genomic electropherotype, and EM observations to confirm the classification of V196/XJ/2014 strain isolated in Xinjiang Uygur Autonomous Region as a member of BTV. However, results from serotype‐specific conventional reverse transcription (RT)‐PCR, real‐time RT‐PCR, and virus neutralization (VN) tests indicated that the virus did not belong to any recognized BTV serotypes. Genomic analysis revealed that the nucleic acid and amino acid sequence homology between V196/XJ/2014 segment 2 and VP2 proteins with other recognized BTV serotypes was less than 63.4% and 61.4% (Figure 4). Additionally, segment 2 formed a separate “nucleotype” in the phylogenetic tree. The findings suggest that V196/XJ/2014 does not belong to any previously reported serotypes of BTV, but represents a novel putative serotype, namely BTV‐29.

The distribution of BTV spans across all continents, excluding Antarctica. As a result of prolonged geographical isolation, BTV strains in various regions have undergone unique gene reassortment to adapt and thrive within their respective local ecosystems. Reassortment events can transpire between different serotypes, attenuated vaccine strains, and wild strains, as well as between locally endemic strains and foreign counterparts. In nature, it is common for different serotypes or topotypes of BTV strains to co‐infect the same host animal, leading to gene reassortment on any segment and resulting in a significant level of genetic diversity within BTV [52]. The advances of gene sequencing technology in recent years have gradually led to the acceptance of BTV typing through sequence analysis, making it an essential tool for isolate traceability. Particularly in cases of virus reassortment, whole‐genome sequencing plays a crucial role. The nucleotide sequences of structural proteins (VP1, VP3, VP4, VP6, VP7) and non‐structural proteins enable the classification of most BTV isolates into distinct Eastern or Western topotypes, which can be further divided into various geographic subgroups. The Eastern topotype encompasses isolates from Indonesia, Malaysia, India, China, Japan, and Australia, while the Western topotype comprises isolates from Africa, North and South America [20, 53].

Numerous studies have demonstrated a high frequency of reassortment events among gene segments of different BTV serotypes and topotypes in China. For example, Seg‐1, ‐2, ‐3, ‐4, and ‐6 of the BTV‐7 GD008 strain isolated from Guangdong Province belong to the Western topotype and exhibit high similarity (>90.5%) with the South African strain. On the other hand, Seg‐5, ‐7, ‐8, ‐9, and ‐10 of this strain are classified as Eastern topotype and show significant reassortment (>95.4%) to BTV‐1 (Y863) isolated in 1979 and YTS4 strains of BTV‐4 in China. These findings confirm a genetic reassortment event between an African strain and the prevalent Chinese BTV strain [54]. Additionally, it was observed that the Seg‐6 gene of the Chinese BTV‐24 strain had undergone genetic reassortment with that of the South African BTV‐10 strain resulting in VP2 protein from BTV‐24 being combined with VP5 protein from BTV‐10 to form the outer capsid structure of BTV‐24 [55].

## 4. Insect Vector—*Culicoides*

The *Ceratopogonidae* is a family of insects belonging to the order Diptera, compassing a diverse range of species with a global distribution. There are 6502 known species of *Ceratopogonidae*, which are classified into five subfamilies and 133 genera [56]. In China, there have been reports of four subfamilies, 39 genera, and 1015 species [57]. Blood‐sucking midges, belonging to the family *Ceratopogonidae*, are a group of insects that feed on the blood of humans, livestock, and other animals. There are 1834 species of blood‐sucking midges distributed across four genera worldwide [58]. These four genera include *Culicoides*, *Lasiohelea*, *Leptoconops*, and *Austroconops*. Among these, *Culicoides* is the largest genus within the Ceratopogonidae family. A total of 414 species of blood‐sucking midges, belonging to the genera *Culicoides*, *Lasiohelea*, and *Leptoconops*, have been documented in China [59]. *Culicoides*, measuring only 1–3 mm in size [60], are widely distributed across all continents except Antarctica. Currently, *Culicoides* is the genus with the highest number and species of blood‐sucking midges known to date. There are currently 1399 known species of *Culicoides* belonging to 35 subgenera worldwide [61], while China has identified 480 species of *Culicoides* within 12 subgenera [57].

**Figure 5:**
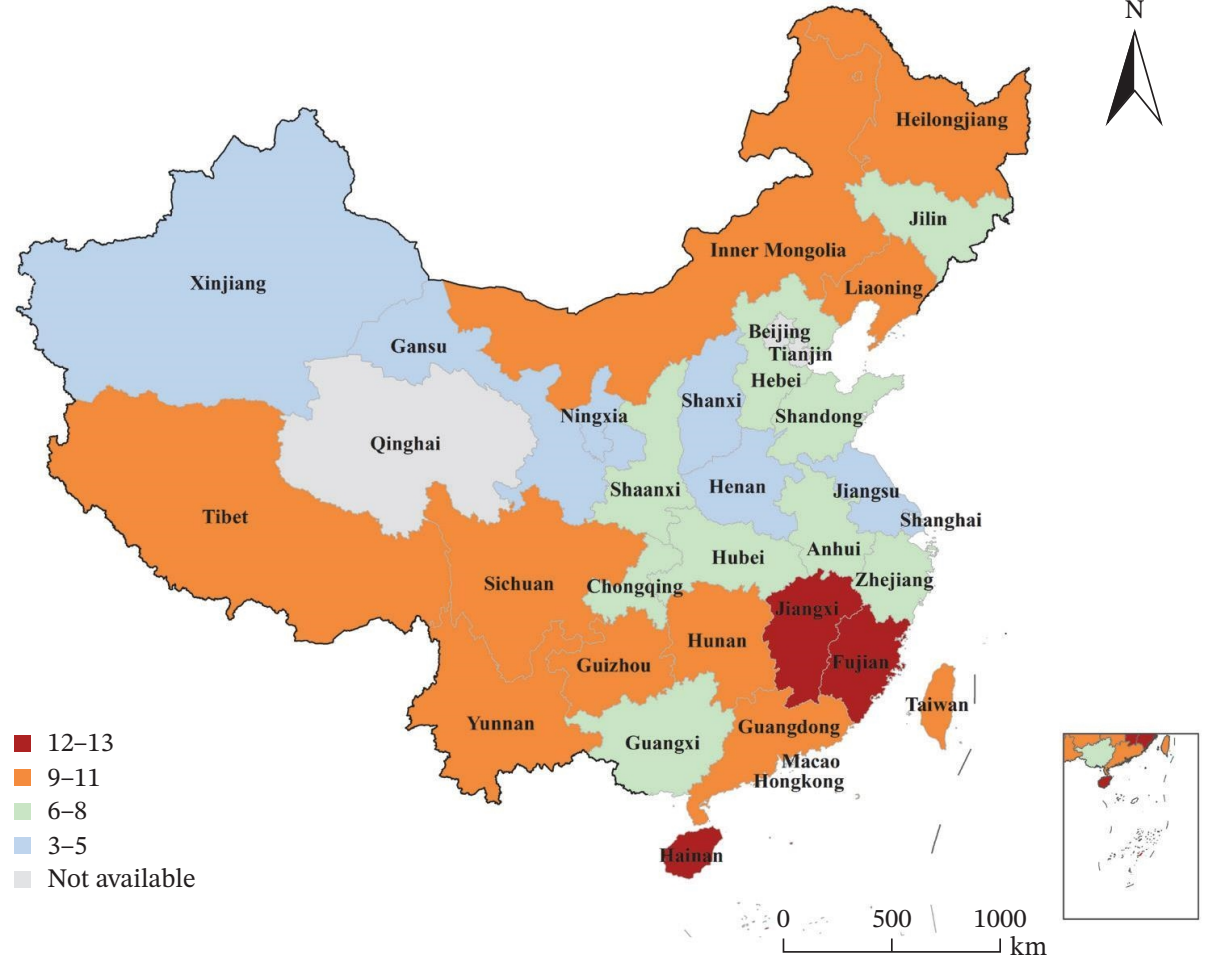
The species and its distribution of *Culicoides* that transmit or potentially transmit BTV in China. Data from Li and Wang [62].

*Culicoides* serve as vectors for a variety of viruses, with at least 42 *Culicoides* species identified as potential transmitters or carriers of animal viruses [62, 63]. Once infected, *Culicoides* maintain their virulence throughout their lifespan [64]. Currently, more than 50 viruses have been isolated from *Culicoides* [65], including BTV, Schmallenberg virus (SBV), African horse sickness virus (AHSV), vesicular stomatitis virus (VSV) and others [66]. To date, ~32 species of *Culicoides* have been implicated in the transmission of BTV, out of which 7 are considered significant vector species due to their natural ability to carry or successfully infect BTV under laboratory conditions [59]. Regional variations exist in the species of *Culicoides* that transmit BTV. For instance, *C. imicola*, *C. obsoletus*, and *C. schulitzei* are the primary vectors in Africa and the Middle East, while in Australia, it is mainly transmitted by *C. brevitarsis*, *C. wadai*, *C. fulvus*, and *C. actoni*. In North America, it is primarily spread by *C. variipennis* and *C. insignis*; whereas in Europe, it is predominantly transmitted by *C. imicola* [67]. In China, 18 *Culicoides* species have been confirmed to effectively transmit BTV, and these species are widely distributed across the country [62] (Figure 5).

The distribution pattern of *Culicoides* capable of transmitting or potentially transmitting BTV is consistent with the detection of BTV in all provinces, autonomous regions, and municipalities directly under the Central Government except Beijing, Tianjin, Hong Kong, and Macao. The abundance of blood‐sucking midges is correlated with species dominance, whereby areas rich in species exhibit less apparent dominance of a single species, while areas with lower species richness often display clear distribution patterns of dominant species [68]. The community diversity analysis revealed a prominent distribution pattern of dominant midge species from south to north, with a higher diversity index observed in the southern regions compared to the northern ones. *C. homotomus* emerged as the most widely distributed species, being found in 29 provinces and autonomous regions.

The first literature on blood‐sucking midges, describing the life history and blood‐feeding habits of *Culicoides*, was published in 1713 [41]. The family *Ceratopogonidae* was established by Maloch (1914–1917), who independently distinguished it from *Chironomidae* in 1917 after studying American midges [69]. The study of *Ceratopogonidae* in China commenced relatively late. In recent decades, significant advancements have been made in the research on *Culicoides* in China. However, there remains a lack of comprehensive investigation into *Culicoides*, including insufficient background knowledge and an ambiguous transmission mechanism. The Viriomics technology enables accurate, high‐throughput, and unbiased sequencing of viruses in samples and has been extensively utilized [70, 71]. Currently, studies on insects using viriomics are primarily limited to mosquitoes and ticks, with very few investigations conducted on *Culicoides*. Zeng [72] conducted metagenomic sequencing and evolutionary analysis of viruses carried by *Culicoides* in the Yunnan border area, revealing that the prevalence of BTV from *Culicoides* in 2020 was 15.79%, which closely resembled the prevalence rates of BTV from cattle in 2019 (16.03%) and 2021 (23.22%) within the same region. Furthermore, viromic studies demonstrated that *Culicoides* in Yunnan Province predominantly harbored viruses belonging to seven families, including *Chuviridae*, *Flaviviridae*, *Herpeviridae*, and *Retrovirida*. *C. luteolus* is a newly discovered species of *Culicoides* found in Indonesia and Malaysia [73]. Yang et al. [74] initially identified this female specimen in Hekou County, located on the southern border of Yunnan Province, while Feng et al. [75] collected female *C. luteolus* specimens in Lushui, Fugong, and Gongshan counties situated along the China‐Myanmar border in the northwest region of Yunnan Province. The study conducted by Liu et al. [73] represents the initial documentation of male *C. luteolus* presence in China, offering valuable insights for future identification endeavors.

Currently, the niche model serves as the primary approach for evaluating species’ response to climate change [76]. The maximum entropy niche model (MaxEnt) within the niche model quantifies the potential geographic distribution of species based on existing distribution data points and determines their maximum entropy survival probability distribution. This capability enables the estimation and prediction of species distribution. It has found extensive application in animal and plant conservation, invasive alien species management, and infectious disease distribution forecasting. Zheng et al. [77] employed MaxEnt to predict the distribution of *C. oxystoma* in China and evaluated the influence of environmental variables on its occurrence. Their study revealed that the most suitable areas for *C. oxystoma* were mainly distributed in northwest China, northeast China and most parts of south China. Among the various environmental factors considered, the available water content in soil (25%), the lowest temperature in the coldest month (18.1%), precipitation in the driest month (18%) and average maximum wind speed (13.2%) emerged as crucial determinants shaping the distribution pattern of *C. oxystoma*. Among them, precipitation in the driest month was the most important environmental factor affecting the distribution of *C. oxystoma* in the model. In a separate investigation into the impact of climatic factors on the distribution of *Culicoides*, Wang et al. [78] observed that there was no significant correlation between altitude (*R* ^2^ = 0.130, *p* = 0.639) or wind speed (*R* ^2^ = 0.059, *p* = 0.641) with the daily collection amount of *Culicoides*; however, a positive correlation was found with precipitation (*R* ^2^ = 0.199, *p* = 0.038), while average temperature exhibited a negative correlation (*R* ^2^ = 0.838, *p* = 0.010).

The control of vectors is a fundamental measure for preventing and managing vector‐borne viral diseases. Understanding the species composition and blood‐feeding habits of vectors in various ecological settings serves as a prerequisite and foundation for effective prevention and control strategies. A number of studies [57, 79, 80] have revealed that *C. arakawai*, *C. jacobsoni*, and *C. oxystoma* are the predominant species in residential areas; whereas in cattle farms, the dominant species include *C. parahumeralis*, *C. jacobsoni*, and *C. palpifer*; furthermore, sheep pens and pig pens are primarily inhabited by *C. oxystoma*; finally, chicken pens and dog pens exhibit a dominance of *C. arakawai*. Additionally, *C. arakawai*, *C. oxystoma*, *C. punctatus*, and *C. homotomus* all exhibit hematophagy in both humans and animals, with each species displaying the tendency to feed on 2–4 different animal hosts simultaneously. These studies serve as valuable references for further investigations into the ecological behaviors of blood‐feeding midges and diseases transmitted by these insects.

## 5. Detection Methods

The utilization of various detection methods is essential for the determination and effective management of BT, playing a pivotal role in its prevention and control. The detection methods for BTV primarily encompass virus isolation, specific serological testing, and viral nucleic acid detection.

### 5.1. Detection Methods Targeting BTV Antigen

#### 5.1.1. RT‐PCR and qRT‐PCR

Chinese researchers have conducted extensive research on methods for detecting BTV, with a primary focus on nucleic acid detection. Specific RT‐PCR and qRT‐PCR methods were developed for detecting BTV‐29 (V196/XJ/2014 strain) [81]. The sensitivity for nucleic acid of BTV‐29 was 1.12 × 10^2^ copies/μL for RT‐PCR and 1.12 × 10^1^ copies/μL for qRT‐PCR, respectively. Importantly, both BTV‐29‐specific RT‐PCR and qRT‐PCR did not exhibit any cross‐reaction with BTV‐1 to BTV‐24, Epidemic hemorrhagic disease virus (EHDV), Palyam virus (PALV), or Akabane virus (AKAV). The one‐step RT‐PCR and nested RT‐PCR assays were developed by Feng [82]. The one‐step RT‐PCR method can effectively detect BTV‐1 to BTV‐24 with a detection limit of 100 TCID_50_ without any cross‐reaction with related viruses. Additionally, the nested RT‐PCR strategy exhibited the capability to detect BTV serotypes 1–24 and 26 with a detection limit of 10 TCID_50_ while maintaining specificity against related viruses. The multiplex PCR technology is developed based on PCR, enabling simultaneous detection of multiple pathogens in a single reaction. In comparison to single PCR, multiplex PCR not only maintains excellent specificity and sensitivity but also demonstrates significant superiority in detecting mixed viral infections. Chinese researchers have developed a range of nucleic acid detection technologies, including multiplex PCR, RT‐PCR, and qRT‐PCR. The study conducted by Li et al. [83] established a serotype‐specific real‐time RT‐PCR method for the detection of 12 serotypes of BTV (BTV‐6, ‐8, ‐10, ‐11, ‐13, ‐14, ‐17, ‐18, ‐19, ‐20, ‐22, and ‐23). Additionally, they developed a serotype‐specific RT‐PCR method for the identification of the prevalent BTV serotypes in China (BTV‐1, ‐2, ‐3, ‐4, ‐5, ‐7, ‐9, ‐12, ‐15, ‐16, ‐21, and ‐24) [84]. These methods provide reliable tools for the detection, diagnosis, and molecular epidemiological investigation of BTV in China.

#### 5.1.2. RT‐Loop‐Mediated Isothermal Amplification (RT‐LAMP)

A BTV RT‐LAMP method was established by Li et al. [85], which demonstrates specific detection of BTV nucleic acids belonging to the 12 epidemic serotypes in China (BTV‐1, ‐2, ‐3, ‐4, ‐5, ‐7, ‐9, ‐12, ‐15, ‐16, ‐21, and ‐24), without any cross‐reaction with nucleic acids from EHDV, AKAV, AHSV, Chuzan disease virus (CHUV), and foot‐and‐mouth disease virus (FMDV). The lower limit of detection for this method is 4.5 copies/μL of BTV genomes. It enables completion of the amplification reaction within 45 min.

#### 5.1.3. Bio‐Bar Code Assay (BCA) and Fluorescent Quantitative (FQ)‐BCA

Zhang [86] developed BCA for BTV and the FQ‐BCA for BTV. The BCA is a novel labeled immunoassay technology that was first reported in 2003 by a research group led by American scientist Mirkin. It is characterized by its high sensitivity. The basic principle is similar to the highly specific capture of antigens using the double antibody sandwich method in ELISA detection, with the indirect detection of antigen substances achieved through the detection of specific bar code DNA strands. Conventional PCR amplification or microarray detection based on gold label silver staining is used for detecting specific barcode DNA strands. Both the BCA and FQ‐BCA methods demonstrated excellent specificity and repeatability within and between batches, and the FQ‐BCA method displayed superior repeatability.

In addition, other BTV detection methods, including Antigen capture ELISA (AC‐ELISA) method [87], nanoprobe detection method [88], liquid chip method [89], real‐time fluorescent RT recombinase‐aided amplification (RT‐RAA) method [90], genomeLab eXpress profiling polymerase chain reaction (GeXP‐PCR) [91], have been established. These detection methods have good specificity, sensitivity, and stability, making them suitable for BT detection.

### 5.2. Detection Methods Targeting BTV Antibody

The commonly employed methods for detecting BTV antibody primarily encompass enzyme‐linked immunosorbent assay (ELISA), VN, and agar gel immunodiffusion (AGID).

#### 5.2.1. ELISA

ELISA is a quantitative test capable of detecting the protein and antibody valence content in a sample, exhibiting characteristics such as high throughput, simplicity, ease of application, and widespread popularity. Being exclusively present in BTV‐infected cells and not incorporated into virions, the level of antibodies against NS3 differs between animals vaccinated with inactivated vaccines and naturally infected animals [92]. Huang et al. [93] developed an indirect ELISA (I‐ELISA) method utilizing recombinant NS3 protein as the coated antigen. This method exhibits a detection sensitivity of 1:1280 and demonstrates excellent repeatability with a coefficient of variation (CV) within and between batches below 10%. Comparative analysis against commercial BT ELISA kits revealed a perfect coincidence rate of positive BT samples. Consequently, the developed indirect ELISA could distinguish the immuned animals from infected animals of BTV. Miao et al. [94] developed a group‐specific blocking ELISA (B‐ELISA) method for BT by preparing BTV group‐specific polyclonal antibodies. The sensitivity, specificity, and repeatability of this detection assay have been demonstrated. The antibody titers detected by the blocking‐ELISA and VNT were similar with no significant differences observed (correlation value: 0.898). There is limited literature available on the utilization of core‐like particles (CLP) as coated antigens for the detection of serum antibodies against BTV. A B‐ELISA method was developed using recombinant CLP derived from BTV‐16 [95]. The method demonstrates excellent specificity and repeatability, exhibiting no cross‐reactivity with positive sera of AHSV, EHDV, FMDV, and VSV. The CV for both intra‐ and inter‐assay precision tests is less than 10%. Furthermore, the established method exhibits a high level of agreement (97.8%) when compared to a commercial ELISA kit for the detection of BTV antibodies. The Competitive ELISA (C‐ELISA) is a serological diagnostic method recommended by WOAH for detecting BTV antibodies. Numerous studies on C‐ELISA methods have been reported in China [96, 97]. Miao et al. [98] developed a group‐specific polyclonal antibody against BTV and optimized antigen fixation technology to establish a C‐ELISA method. A total of 150 known negative sera and 55 known positive sera were tested using the VN test, C‐ELISA BTV antibody detection kit produced by VMRD, and the established C‐ELISA method, respectively. The results demonstrated that the established C‐ELISA method exhibited a coincidence rate of 100% with the VN test and a coincidence rate of 96.7% with the imported commercial kit. This cost‐effective method can be utilized for screening serum antibodies against BT.

#### 5.2.2. Colloidal Gold Immunochromatography Test Strip

In addition to the ELISA method, Wang et al. [99] developed a colloidal gold immunochromatography test strip. As a result, even when diluted at a ratio of 1:64, positive sera for bovine BT could be accurately detected. The prepared colloidal gold strip exhibited no cross‐reactivity with sera from other related diseases. When compared to the foreign commercial kit, the coincidence rate between the strip and the commercial ELISA kit (IDEXX) was found to be 92.7% (51/55). Furthermore, the entire testing process can be completed within just 10 min.

#### 5.2.3. AGID

The AGID method necessitates a substantial quantity of antigens at a high concentration for effective detection, and its sensitivity is relatively limited. The AGID method used for BTV detection was studied by Chinese scholars in 1989 [100], and since then, there have been limited reports on the investigation of this technique. Some of the detection methods for BTV established by Chinese researchers are shown in Table 2.

**Table 2:** BTV detection methods established in China.

Methods	Targets	Sensitivity
RT‐PCR [81]	Nucleic acid of BTV‐29 (V196/XJ/2014 strain)	1.12 × 10^2^ copies/μL
qRT‐PCR [81]	Nucleic acid of BTV‐29 (V196/XJ/2014 strain)	1.12 × 10^1^ copies/μL
qRT‐PCR [83]	Nucleic acid of BTV‐6, ‐8, ‐10, ‐11, ‐13, ‐14, ‐17, ‐18, ‐19, ‐20, ‐22, and ‐23	12 copies/μL (BTV‐8) to 57 copies/μL (BTV‐14)
RT‐PCR [84]	Nucleic acid of BTV‐1, ‐2, ‐3, ‐4, ‐5, ‐7, ‐9, ‐12, ‐15, ‐16, ‐21, and ‐24	1.28 × 10^2^ copies/μL (BTV‐2) to 9.62 × 10^2^ copies/μL (BTV‐1)
RT‐LAMP [85]	Nucleic acid of BTV‐1, ‐2, ‐3, ‐4, ‐5, ‐7, ‐9, ‐12, ‐15, ‐16, ‐21, and ‐24	4.5 copies/μL
Real‐time fluorescent reverse transcription recombinase‐aided amplification (RT‐RAA) method [90]	Nucleic acid of BTV‐8	20 copies/μL
GenomeLab eXpress Profiling polymerase chain reaction (GeXP‐PCR) [91]	Nucleic acid of BTV	10^2^ copies/μL
Bio‐bar codes assay (BCA) [86]	VP7 protein of BTV	1 fg/mL for the conventional PCR detection and 10 fg/mL for the chip detection
	BTV	10^−4^ TCID_50_ for the conventional PCR detection and 10^−3^ TCID_50_ for the chip detection
Fluorescent quantitative bio‐bar codes assay (FQ‐BCA) [86]	VP7 protein of BTV	100 ag/mL
	BTV	10^−5^ TCID_50_
Antigen capture ELISA (AC‐ELISA) [87]	BTV	10^2.79^ TCID_50_
Nanoprobe detection method [88]	VP7 protein of BTV	10^−2^ fg/mL
Indirect ELISA (I‐ELISA) [93]	Antibodies against BTV	1:1280 dilution of BTV standard positive serum
B‐ELISA [95]	Antibodies against BTV	1:328 dilution of BTV standard positive serum
Colloidal gold strip assay [99]	Antibodies against BTV	1:64 dilution of BTV standard positive serum

## 6. Vaccines

At present, there are no effective preventive or therapeutic drugs against BTV, and immunization continues to be one of the most efficient approaches to control this disease.

### 6.1. Attenuated and Inactivated Vaccines

Chinese scholars have conducted some studies on attenuated and inactivated BT vaccines. Zhang et al. [101, 102] first reported the occurrence of BT in China and subsequently conducted research on chicken‐embryo‐adapted attenuated vaccine and inactivated vaccine based on the isolation and identification of the BTV. These vaccines played an important role in the control of the BT outbreak at that time. In one study, an inactivated vaccine was prepared using V863 strains of BTV‐1, which is capable of inducing the production of neutralizing antibodies in sheep [103]. The recommended dosage during production is 10 μg of viral antigen per animal, and an antibody titer exceeding 64 can be considered as the basis for effective research and development of future BTV‐inactivated vaccines. The selection of an inactivated agent and adjuvant is crucial for the development of an inactivated vaccine. During preliminary development of a BTV‐8 inactivated vaccine, it was discovered that ISA15VG exhibited promising potential as an adjuvant candidate, whereas hydroxylamine hydrochloride and β‐propiolactone (BPL) demonstrated effective viral inactivation properties [104]. Considering factors such as labor cost, ease of operation, and immune effect, it is recommended to employ BPL for virus inactivation alongside ISA15VG as the preferred adjuvant.

### 6.2. Vaccines Based on BTV Reverse Genetics System

The immune protection effect and procedures of inactivated and attenuated vaccines are serotype‐specific, posing complexities in areas with multiple prevalent serotypes. Moreover, the attenuated BT vaccine exhibits teratogenicity and potential reassortment with wild strains to generate novel genotypes, leading to a range of biosafety concerns [105]. The development of novel multivalent, safe vaccines with the capability to differentiate infected from vaccinated animals (DIVA) is of paramount importance. The BTV reverse genetic system, established by Boyce and Roy [106], has facilitated research on virus mutation [107] resulting from BTV gene reassortment and the development of new vaccines [108]. Yang [109] successfully established the first‐ever reverse genetic operating system for BTV in China and effectively generated BTV. The rescued virus rBTV1 exhibited a growth curve similar to that of the native strain SZ97/1, reaching a peak virus titer of 1 × 10^8^ TCID_50_/mL within 60–72 h post‐infection.

Multiple BTV serotypes circulate in China, with significant variations in pathogenicity among them. For example, the BTV‐16 exhibits high pathogenicity in sheep, while the BTV‐4 shows low pathogenicity [110]. The VP2 and VP5 proteins encoded by Seg‐2 and Seg‐6 not only determine the virus’s serotype but also play a crucial role in specific virus‐cell adsorption and virion assembly [111, 112]. The reverse genetic system of BTV‐16, based on the highly pathogenic BTV‐16/V158 strain in China, was successfully established by Li et al. [113]. Subsequently, Seg‐2 and Seg‐6 of the BTV‐16 strain were replaced with corresponding segments from BTV‐4, resulting in the rescue of a novel serotype and proliferative variant named BTV‐16/V158‐RG (BTV‐4/S2, S6). These findings provide a robust technical platform for further genomic analysis of key virulence genes associated with the Chinese BTV‐16 strain and facilitate the development of engineered vaccines. Guo [114] developed a reverse genetic system solely based on plasmids and employed it to rescue the recombinant BTV‐16 carrying the human influenza hemagglutinin (HA) tag in the VP2 protein. The immunogenicity of these recombinant tagged BTV‐16 inactivated vaccines was assessed in mice and sheep. The potential of recombinant BTV‐16 for generating DIVA inactivated vaccines was preliminarily validated.

### 6.3. Virus‐Like Particles (VLPs)

The absence of viral genome in VLPs prevents autonomous replication, thereby mimicking the presentation to immune cells observed during natural virus infection. This characteristic enables them to elicit an immune response almost identical to that induced by the original virus, rendering them advantageous in terms of safety, non‐inactivation, DIVA characteristics, and other aspects. Huang [115] produced VLPs of BTV16 for the purpose of immunizing mice. After two doses of immunization, the titer of specific antibody and neutralizing antibody stimulated by BTV16 VLPs were 1:128 and 1:64, respectively, indicating that BTV16 VLPs have potential as a candidate vaccine for BTV16.

### 6.4. Live Carrier Vaccines

The use of live carrier vaccines effectively prevents the transmission of live attenuated vaccines to the natural environment and reduces the risk of gene recombination. It also overcomes the limitations of multiple high‐dose immunizations required for inactivated vaccines, making it an important direction for BT vaccine development. Zhang et al. [116] successfully integrated the BTV16 VP2 gene into the recombinant vaccinia virus genome, resulting in the creation of rVTT‐VP2, a recombinant BTV16 VP2 vaccinia virus. This recombinant virus had no significant impact on mouse growth but demonstrated effective stimulation of T lymphocyte differentiation and splenic lymphocyte proliferation, as well as induction of specific immune responses in BALB/C mice. The results suggest that rVTT‐VP2 has high safety and can effectively stimulate the specific response of the body and can be further used in the follow‐up vaccine research.

Currently, there are no mandatory state requirements for BT immunization, and relevant enterprises and scientific research institutions lack sufficient motivation to develop vaccines. Despite extensive vaccine research in China, commercial application remains limited.

## 7. Recommendations for BT Prevention and Control

In recent years, with the implementation of major national strategies such as the “Belt and Road Initiative” in China, the personnel exchanges and trade between China and other countries have become increasingly frequent. Coupled with global warming, the distribution range of insect vectors has expanded, and virus recombination occurs frequently. Against this backdrop, it is even more necessary to enhance the awareness of the harm of BT and carry out corresponding prevention and control work to avoid causing significant losses to China’s animal husbandry as much as possible.

### 7.1. Enhance Public Awareness Regarding the Hazards Associated With BT

Positive samples of the pathogen or antibodies have been detected in multiple provinces in China. Notably, mandatory immunization against BT has not yet been mandated, resulting in generally low immunity to the disease among domestic animals such as cattle and sheep. The key vector in the transmission process of BT, *Culicoides*, is widely distributed in China. Once the BTV in China undergoes genetic variation or recombination, it can form a new “infection source‐vector‐susceptible animal” transmission chain, posing a potential risk of BT outbreak.

In June 2022, the Ministry of Agriculture and Rural Affairs of China reclassified BT from a Class I to a Class II animal disease (http://www.moa.gov.cn/govpublic/xmsyj/202206/t20220629_6403635.htm, accessed on October 01, 2023). With the decrease in reported cases of animal mortality caused by BT in recent years, there is a potential decline in public awareness towards this disease. Nevertheless, it remains crucial for governments at all levels, animal disease prevention and control departments, relevant practitioners to maintain their attention and support in order to effectively control and prevent the spread of BT. However, considering the historical context of BT outbreaks, the BT that occurred in Spain and Portugal in 1956 resulted in the mortality of 179,000 sheep, with a staggering mortality rate of 75% [117]. Similarly, when the BT first emerged in China in 1979, it caused significant economic losses due to a substantial number of susceptible animals succumbing to the epidemic at its early stages. The BTV exhibits numerous serotypes, and the genes of these distinct serotypes readily undergo mutation and recombination, thereby rendering the viral epidemic outcomes unpredictable. For instance, the BTV‐8 virulent strain that circulated in Europe in 2006 displayed significant divergence from its predecessor, as it not only induced evident clinical symptoms in infected sheep but also exhibited pathogenicity towards cattle. Prior to this occurrence, cattle were considered as recessively infected animals [44].

Although there have been no reports of significant economic losses caused by BT in China in recent years, it is still necessary to pay closer attention to the disease and accurately grasp the basic situation of its epidemic distribution, as well as the distribution and transmission ability of insect vectors. Corresponding basic research and necessary technical reserves should be carried out to effectively respond to emergencies. In addition, even if animals infected with BTV do not show clinical symptoms, their growth performance will decline and feed conversion ratio will decrease, leading to substantial economic losses for the livestock industry.

### 7.2. The Adoption of Integrated Prevention and Control Technology System

The complexity of BT infection, as a vector‐borne disease, necessitates the implementation of multiple control techniques. This is one of the reasons why the WOAH designates it as a notifiable infectious disease. Comprehensive technical measures, such as serological surveillance, prompt identification, isolation and disposal of infected animals in farms, particularly breeding farms; restriction of animal movement in epidemic areas; placement of sentinel animals in key epidemic zones; elimination of blood‐sucking insects like *Culicoides*, mosquitoes and ticks during the outbreak season; reduction of grazing activities during peak *Culicoides* activity hours; and environmental disinfection should be implemented to minimize the impact of BT. In Xinjiang and Inner Mongolia, the pastoral areas mainly raise sheep and have long maintained the nomadic habit. Therefore, it is even more necessary to take necessary measures to reduce the contact between livestock and *Culicoides* during the peak season of *Culicoides*. In addition, the neighboring countries in the southwest bordering China have relatively serious cases of BT disease in cattle. Therefore, it is necessary to strengthen the quarantine of inbound animals to prevent the disease from being introduced into China from abroad. The infection of BT in cattle and goats is predominantly persistent or recessive, often lacking characteristic clinical symptoms, which poses challenges for epidemiological monitoring and prevention efforts. Given the absence of a mandatory immunization plan against BT in China, it is imperative to enhance long‐term and systematic surveillance for effective prevention and control. Due to the absence of cross‐immunity between different serotypes of BTV, accurate determination of the local BTV serotype is imperative for effective prevention and control measures. Currently, the epidemiological investigation of BTV in China lacks systematicity, with varying quantities and qualities of reported data across regions. Furthermore, a significant proportion of serological investigations only focus on detecting BTV serogroup‐specific antibodies without providing specific information about prevalent serotypes in local areas. Despite the isolation of 17 BTV serotypes in China, the possibility of other unidentified serotypes cannot be disregarded.

Australia’s BTV surveillance system can serve as a valuable reference for us. In the 1970s, Australia initiated the establishment of a national arbovirus surveillance system, which involved monitoring BTV through the deployment of sentinel animals. This comprehensive approach enabled them to gain a thorough understanding of the distribution and activity of BTV and its vectors in Australia, as well as timely detection of new invasive foreign arboviruses. Surveillance sites and sentinel animals play a crucial role in early warning of BT, risk assessment, epidemiological research, and virus isolation. Selecting cattle and sheep aged between 6 months and 1 year with no BTV antibodies as sentinel animals may enhance their susceptibility to the disease. Moreover, it is essential to raise and manage these sentinel animals alongside local susceptible animals without using insecticides. This approach increases the likelihood of vector insects biting the sentinels and consequently enhances their chances of natural BT infection.

The regionalized management measures implemented by the European Union can also serve as a valuable reference for China’s BTV prevention and control practices. Guided by the core concept of constructing the entire region as a BTV‐free zone, the EU has established a comprehensive prevention and control system through the delineation of restricted areas and surveillance zones. Differentiated management strategies are applied to the inside and outside of these restricted areas, which include the implementation of stringent regulations on the movement of animals and animal reproductive materials, the joint execution of serological, clinical, and vector monitoring programs with multiple stakeholders, as well as the establishment of platforms for the notification and exchange of BTV monitoring and surveillance information. The aforementioned prevention and control strategies are concurrently disseminated as EU directives, resolutions, and regulations with obligatory legal implications, consistently supplemented and enhanced to mandate their implementation by member states. These measures implemented by the EU have effectively averted and managed the BT epidemic within its borders, thereby offering valuable insights for China’s own efforts in preventing and controlling BT.

### 7.3. Further Research on Prevention and Control of BT

In China, there is a wealth of literature on antibody surveillance, virus isolation, identification, and detection methods for BTV; however, limited research has been conducted on therapeutic drugs, vector control measures, and early warning models.

#### 7.3.1. BTV Overwintering Mechanisms

Overwintering is the underlying factor contributing to the prolonged persistence of BTV in specific regions. In numerous temperate areas, adult *Culicoides* vector activity and BTV replication come to a halt during winter, resulting in an almost complete interruption of virus transmission. However, BT outbreaks frequently resurge several months after this “Silent Period” surpassing the typical viremia duration in ruminants or lifespan of adult vectors [118]. Investigating BTV overwintering mechanisms is therefore critical for effective prevention and control.

#### 7.3.2. BT Vector Research

Comprehensive research on BTV vectors in China is lacking, particularly regarding vectors responsible for BT transmission. While Culicoides is internationally recognized as the primary BT vector, reports on how Culicoides transmits BTV and whether other blood‐sucking insects contribute to transmission remain scarce. Nevertheless, certain studies have observed seroconversion of BTV antibodies even during seasons when *Culicoides* activity is absent [119], suggesting the possibility of alternative insect vectors contributing to disease transmission. It is of paramount practical significance to conduct comprehensive research on the vectors of BT and ascertain the types as well as distribution abundance of these vectors across different regions in China.

#### 7.3.3. Vaccine and Drug Development

Conventional inactivated and attenuated vaccines provide limited protection for domestic animals. While progress has been made in developing new recombinant vaccines, they are not yet ready for commercialization. Specific drugs for BT are not yet available, thus emphasizing the need to enhance research on preventive and therapeutic measures against BT is crucial from both veterinary and economic perspectives. Few studies have been reported on drugs for the prevention and treatment of BT in China. Chen et al. [120] discovered that Tubacin, a specific inhibitor targeting histone deacetylase 6 (HDAC6), exhibits a certain degree of inhibition against BTV‐13 infection. Lv et al. [121] discovered that the administration of early autophagy inhibitor 3‐MA and late inhibitor CQ significantly impeded BTV‐1 replication when treating cells or interfering with Beclin 1, a pivotal autophagy gene. Xing et al. [122] elucidated the precise regulatory mechanism of BTV replication through miRNA in both vector and host cells, thereby establishing a foundation for further identification of potential anti‐BTV drug targets.

#### 7.3.4. Epidemiological Modeling and Early Warning

China has established a quarantine and supervision system for BT; however, there remains a certain disparity when compared to developed countries. In China, there is limited research on the evaluation and early warning of BT. Regarding epidemic risk analysis, qualitative analysis is predominantly employed in China, leading to subjective conclusions. Due to the low level of China’s epidemic early warning, achieving accurate and forward‐looking early warning is challenging. As a non‐endemic country for BT, epidemiological modeling serves as an effective means to study potential outbreaks. With the acceleration of globalization, infectious diseases exhibit obvious spatial heterogeneity, making spatial epidemiology widely applicable in this field. It is imperative to enhance the utilization of spatial epidemiology for conducting predictive analysis and research on the distribution, temporal trends, and spatial characteristics of BT. This will facilitate risk prediction and early warning systems for BT, thereby enabling a comprehensive understanding of its transmission dynamics and patterns. Moreover, it will furnish scientific foundations and timely intelligence information to inform prevention and control strategies. By judiciously allocating human, material, and financial resources, we can effectively curb the epidemic’s progression while minimizing losses.

## 8. Conclusions and Prospects

This paper systematically reviews the distribution of BTV and its vector *Culicoides* in China, as well as the progress in detection methods and vaccine research, and puts forward control suggestions based on practical situations. To date, 17 BTV serotypes have been identified in China, and *Culicoides* are widely distributed. The existing technology has laid a foundation for prevention and control, but the mechanism of the impact of climate on the ecology of vectors needs to be further explored. In the future, it is necessary to deepen the research on the interaction of “virus ‐ vector ‐ host,“ clarify the impact of climate on *Culicoides*; optimize detection technology and improve the screening efficiency of multiple serotypes; accelerate the research and development of multivalent vaccines and enhance cross‐protection. Simultaneously, it is essential to strengthen cross‐regional joint prevention and control, enhance the industry’s awareness of the hazards of BT, and through the construction of a comprehensive technical system, provide a more solid guarantee for the safety of animal husbandry.

## Author Contributions

Diangang Han and Jun Ai contributed to the study design, project administration, and funding acquisition; Jige Xin, Xincheng Ji, Zhigang Song, Weidong Zuo, Shanglian Yin, and Yong Peng contributed to data analysis; Jige Xin and Diangang Han drafted the manuscript; Jige Xin, Xincheng Ji, Zhigang Song, and Miao Ren revised the manuscript and supervised the study.

## Funding

This work was supported by the Expert Workstation of Yunnan (202505AF350101), National Key Research and Development Program of China (2022YFC2601605), Yunnan Key Laboratory of Veterinary Etiological Biology (202449CE340019), the Program of Yunnan High‐level Talents to Young Talents (YNWR‐QNBJ‐2020‐154).

## Disclosure

The funders have no involvement in the design of this review, neither in the collection, management, analysis and interpretation of the data, preparation of the manuscript nor decision to publish. All authors reviewed and edited the final version of the manuscript.

## Conflicts of Interest

The authors declare no conflicts of interest.

## Data Availability

All data are presented within the paper.
